# Large vestibular schwannoma treated using a cranial nerve sparing approach with planned subtotal microsurgical resection and stereotactic radiosurgery: meta-analysis and International Stereotactic Radiosurgery Society (ISRS) practice guidelines

**DOI:** 10.1007/s11060-025-04990-6

**Published:** 2025-04-02

**Authors:** Constantin Tuleasca, Rupesh Kotecha, Arjun Sahgal, Antonio de Salles, Laura Fariselli, Ian Paddick, Jean Régis, Jason Sheehan, John H. Suh, Shoji Yomo, Marc Levivier

**Affiliations:** 1 Neurosurgery Service, Lausanne University Hospital (CHUV), Rue du Bugnon 44-46, BH-08, CH-1011 Lausanne, Switzerland; 2 Faculty of Biology and Medicine (FBM), University of Lausanne (UNIL), Lausanne, Switzerland; 3 Ecole Polytechnique Fédérale de Lausanne (EPFL, LTS-5), Lausanne, Switzerland; 4 Department of Radiation Oncology, Miami Cancer Institute, Baptist Health South Florida, Miami, FL USA; 5 Department of Radiation Oncology, Sunnybrook Health Sciences Centre, University of Toronto, Toronto, ON Canada; 6 University of California Los Angeles, Los Angeles, CA USA; 7 NeuroSapiens and Rede D’Or São Luiz, Unit of Radiotherapy, Fondazione IRCCS Istituto Neurologico C Besta, São Paulo, Brazil; 8 Department of Neurosurgery, Unit of Radiotherapy, Fondazione IRCCS Istituto Neurologico C Besta, Milan, Italy; 9 Medical Physics Ltd, Queen Square Radiosurgery Centre, London, UK; 10 UMR INSERM 1106, Dept of Functional Neurosurgery, Aix Marseille Université, Marseille, France; 11 Department of Neurosurgery, University of Virginia Health System, Charlottesville, VA USA; 12 Division of Radiation Oncology, Aizawa Comprehensive Cancer Center, Aizawa Hospital, Matsumoto, Japan; 13 University of Medicine and Pharmacy, “Grigore T. Popa”, Iasi, Romania; 14 Department of Radiation Oncology, Taussig Cancer Institute, Cleveland Clinic, Ohio, USA

**Keywords:** Vestibular schwannoma, Combined, Hybrid, Planned subtotal resection, Cranial nerve sparing

## Abstract

**Introduction:**

Stereotactic radiosurgery (SRS) has become a standard of care for small- to medium- size vestibular schwannomas (VS), while the majority of patients with large VS still require microsurgical resection due to potential consequences of long tract and cranial nerve compression, intracranial hypertension or hydrocephalus.

**Methods:**

We performed a systematic review and meta-analysis of the literature specific to planned subtotal resection for large VSs followed by SRS to the residual tumor to inform clinical practice guideline development. The Medline and Embase databases were used to apply the Preferred Reporting Items for Systematic reviews and Meta-Analyses (PRISMA) approach to search for manuscripts reporting outcomes for large VSs treated with this paradigm, with a search end date of June 1st 2023. Crude outcomes were pooled using weighted random effects.

**Results:**

12 series met inclusion criteria reporting on treatment outcomes for 677 patients. Overall tumor control was 89.9% (86.9–92.9%, *p* < 0.001), with tumor stability observed in 43.9% (19.9–68%, *p* < 0.001) and tumor reduction in 39.9% (57–74.2%, *p* = 0.02) post-SRS. Facial nerve functional preservation immediately after microsurgery was 88.0% (82.7–93.3%, *p* < 0.001), improving to 94.4% (91.4–97.4%, *p* < 0.001) at last follow-up. Cochlear functional preservation immediately after microsurgery was 58.8% (33.2–84.4%, *p* < 0.001), decreasing to 57.4% (33–81.8%, *p* < 0.001) at last follow-up.

**Conclusions:**

A cranial nerve sparing approach with planned subtotal microsurgical resection and SRS to the residual tumor achieves high rates of tumor control with highly satisfactory outcome of facial and cochlear functional preservation. Clinical practice consensus recommendations on behalf of the International Stereotactic Radiosurgery Society (ISRS) are also presented.

## Introduction

Vestibular schwannoma (VS) is a benign peripheral nerve sheath tumor of the vestibulo-cochlear nerve. Historically, the surgical goal for VS has been an aggressive strategy of achieving a gross total resection (GTR), to maximize long-term tumor control [1], at the expense of facial nerve deficit and hearing loss. Alternative management options to resection evolved over the years to include observation, stereotactic radiosurgery (SRS), and fractionated stereotactic radiotherapy [2]. Rarely, radiation therapy alone is used as upfront treatment in patients with medical comorbidities precluding microsurgery [2].

Although SRS has become a standard of care for small- to medium-size VS [3–6], the majority of patients with large VS still require microsurgical resection due to potential consequences of long tract and cranial nerve compression, as well as intracranial hypertension or hydrocephalus [7]. The estimated recurrence-free survival rates following GTR and STR at 5, 10 and 15 years are 96%, 82% and 73% versus 47%, 17% and 8%, respectively [8]**.** Given that complications of SRS are related to dose and volume, the risks of cranial neuropathies and transient tumor swelling (pseudoprogression), are potential disadvantages to a non-operative approach for large and/or symptomatic VS [9, 10]. Furthermore, a recent systematic review and meta-analysis [2] identified inferior long-term local control rates following single fraction SRS alone in those tumors > 10 cc. However, traditional gross total resection has the disadvantage of causing facial nerve deficits which classically range in incidence between 30–50% [11–13], and compromises the chance of hearing preservation [11, 12, 14].

Over the past two decades, there have been further developments in surgical technologies such that significant decreases in post-operative neurological deficits have been observed [15]. Furthermore, the intent of microsurgery has shifted towards a sub-total resection, in order to reduce the risk of facial nerve deficits, given the known efficacy of SRS in controlling smaller VS [16]. This treatment strategy, consisting of by the combination of subtotal microsurgical resection followed by SRS, is referred to as combined approach. To date, there has yet to be a critical review of the published literature specific to patients treated with such a combined approach. Therefore, the purpose of this systematic review is to summarize outcomes following combined approaches for VSs, and provide treatment recommendations on behalf of the International Stereotactic Radiosurgery Society (ISRS) guidelines committee.

## Methods

### Systematic review

**Fig. 1:**
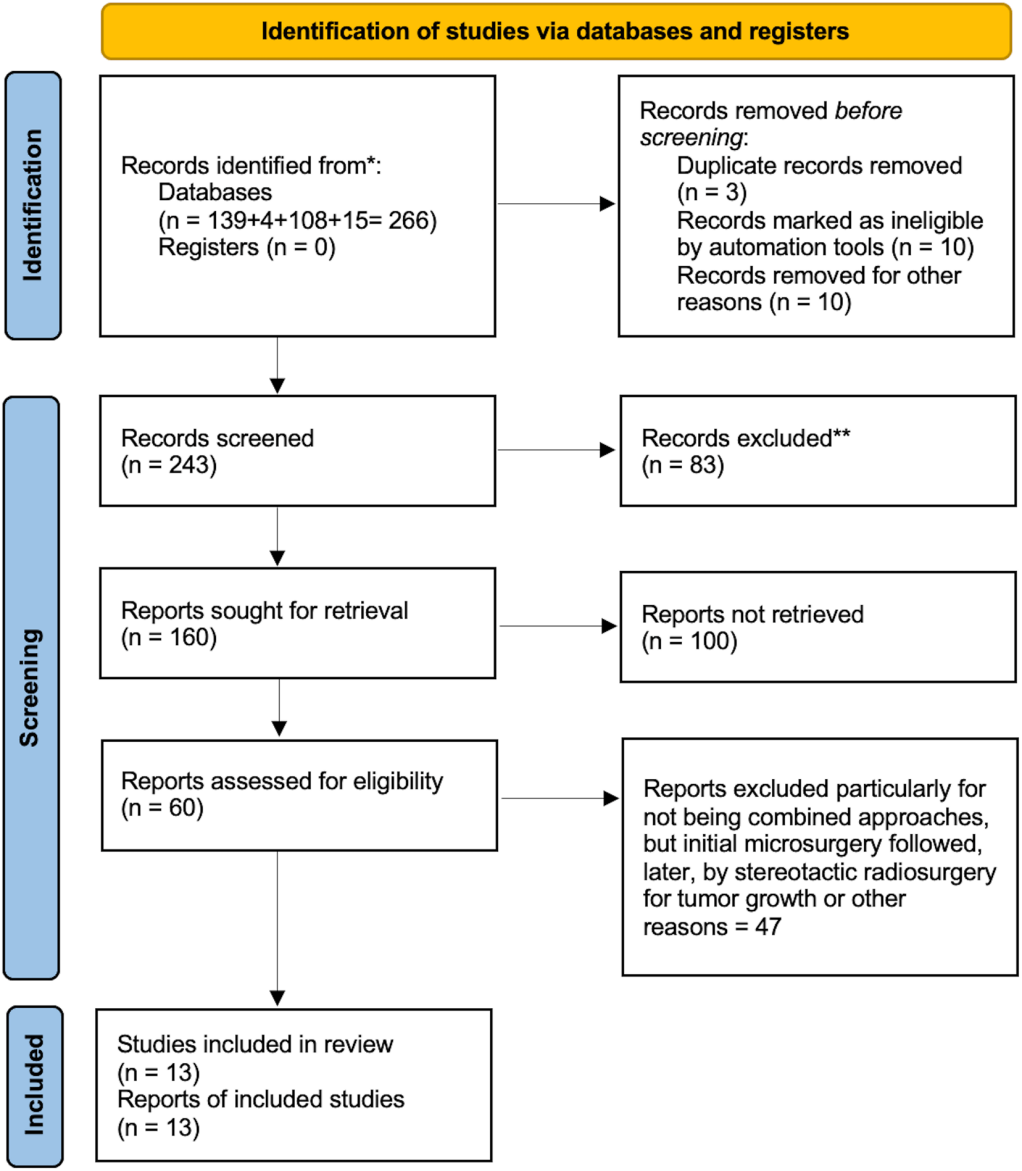
PRISMA flow diagram illustrating the study selection

A systematic review of the literature was performed using the Preferred Reporting Items for Systematic reviews and Meta-Analyses (PRISMA) approach [17] (Fig. 1 and Table 1 summarize demographic data).

**Table 1:** Demographic data for large vestibular schwannomas treated with planned subtotal resection and stereotactic radiosurgery: surgical approach, degree of resection, post-microsurgical complications

References	Number of patients	Criteria and strategy for combined approaches	Age mean (median; range)	Male:female	Follow-up after SRS (months)	NF2-related schwannomatosis from the total number of patients	Surgical approach	Degree of resection	Post-microsurgical complications (other than facial and cochlear)
Iwai et al.[21] (2003)	14 patients	Extrameatal diameter > 30 mm STR + GKS	47(-;18–64)	6:8	32 (-;12–72)	2/14 (14.3%)	R: 13/14 (92.9%) TP: 1/14 (7.1%)	STR: 13/14 (92.9%) PR: 1/14 (7.1%)	Cranial IV palsy: 1/14 (7.1%) Mortality: 0/14 (0%)
Park et al.[25] (2006)	8 patients	diameter > 30 mm STR + GKS	NR	NR	68.8 (-;-)	0/8 (0%)	NR	Radical subtotal removal: 8/8 (100%)	NR
Fuentes et al.[49] (2008)	8 patients	diameter > 30 mm Planned STR + GKS	53(-;24–78)	5:3	46(-;12–73)	NR	R: 8/8 (100%)	STR: 8/8 (100%)	NR
Yang et al.[29] (2008)	61 patients	STR + GKS	-(41; 18–67)	20:41	-(53.7;24.1–102.2)	NR	NR	STR: 61/61 (100%)	NR
Van de Langenberg et al.[28] (2011)	50 patients	Growing Koos grade III and upfront for Koos grade IV Planned STR + GKS	52(-;21–84)	28:22	- (33.8;12–84)	NR	R: 25/50 (50%) TL: 25/50 (50%)	STR: 50/50 (100%)	Hematoma: 2/50 (4%) Hydrocephalus: 2/50 (4%) VP shunt: 1/50 (2%) Lumbar drain: 1/50 (2%) Temporary dysfunction CN IX, X: 3/50 (6%) Transient CN VI: 1/50 (2%) Hemiparesis: 1/50 (2%)
Pan et al.[24] (2012)	G1 (intracapsular decompression):18 patients G2 (extracapsular resection): 17 patients	diameter > 30 mm Planned STR + GKS	G1: 50 ± 3.0 G2: 49 ± 2.3	G1: 10:8 G2: 10:7	G1: 57.7 ± 3.3 G2: 52.7 ± 1.8	NR	G1: R: 18/18 (100%) G2: R: 17/17 (100%)	G1: STR: 18/18 (100%) G2: STR: 17/17 (100%)	G1: 1/18 (5.6%) CSF shunt G2: 1/17 (5.9%) CSF shunt
Daniel et al.[19] (2018)	47 patients	Koos grade IV Planned STR + SRS	51.2(;22–85)	22:25	37.5 (36;0.5–96)	0/47 (0%)	R: 47/47 (100%)	STR: 47/47 (100%)	1/47 (2.1%) transient hypoesthesia after surgery 1/47 (2.1%) CN X deficit 4/47 (8.5%) CSF shunt
Troude et al.[30] (2019)	169 patients (77 upfront SRS)	Koos grade IV STR + GKS	51(-; 16–85)	67:102	Overall: 62 (54; 11–147) 70(75;) in SRS group after SRS (because “67–75” is 95% CI)	10/169 (5.9%)	R: 108/169 (63.9%) TL: 61/169 (36.1%)	GTR: 16/169 (94.7%) NTR: 94/169 (55.6%) STR: 34/169 (20.1%) PR: 15/169 (8.9%)	Keratitis: 20/169 (11.8%); corneal ulcer: 5/169 (2.9%); Abducens: 6/169 (3.6%); IX, X: 5/169 (2.9%); CPA hematoma: 15/169 (8.9%, 10 symptomatic); CSF leak: 18/169 (10.7%); wound infection: 4/169 (2.4%); meningitis: 8/169 (4.7%); hydrocephalus with shunt: 4/169 (2.4%) Pulmonary embolism: 4/169 (2.4%); death: 2/169 (1.2%) 4/169 (2.4%) facial numbness Lower cranial nerve: 5/77 (6.5%), 100% transient
Suero Molina et al.[27] (2019)	160 patients (148 available for clinical, 157 for radiological follow-up)	STR + GKS	-(55;14–89)	63:97	-(36;3–180)	6/160 (3.8%)	1 surgery: 144/160 (90%) 2 surgeries: 11/160 (6.9%) 3 surgeries: 3/160 (1.9%) 4 surgeries: 2/160 (1.1%)	GTR: 118/146 (80.8%) Subcapsular resection: 28/146 (19.2%)	1/160 (0.6%) CSF shunt
Ng et al.[23] (2020)	10 patients	Koos grade IV STR + GKS	47.9 (49.7; 20.6–69.6)	5:5	7.2 (6.9; 1.6–15.5)		R: 8/10 (80%) TL: 2/10 (20%)	STR: 10/10 (100%)	NR
Radwan et al.[31] (2021)	17 patients	Maximum diameter > 40 mm, corresponding to Koos III or IV Planned STR + SRS	56(-;-)	7:15	40 (20,20–128)	NR	NR	STR: 22/22 (100%)	Trigeminal neuropathy: 4/22 (18.2%) 2/22 (9.1%): dysphagia, dysarthria
Iwai et al.[20] (2021)	47 patients	Maximum diameter more than 25 mm	-(60;30–82)	22:25	-(74;24–180)	0/47 (0%) (personal communication)	R: 47/47 (100%)	STR: 47/47 (100%)	1/47 (2.1%) lung abscess 1/47 (2.1%) aseptic meningitis 1/47 (2.1%) pulmonary embolism 1/47 (2.1%) cerebellar venous infarction
Lee et al.[22] (2021)	68 patients	Planned STR + SRS	-(42.5;14–83)	26:42	-(64;25.7–152.4)	0/69 (0%)	R: 66/68 (97%) TL: 2/68 (3%)	STR: 68/68 (100%)	1/68 (1.5%) CSF leakage 1/68 (1.5%) cerebellar dysfunction 1/68 (1.5%) epidural hematoma 1/68 (1.5%)surgical site infection 2/68 (3%) lower cranial nerve dysfunction 3/68 (4.5%) CSF shunt

*STR* Subtotal resection, *SRS* Stereotactic radiosurgery, *GKS* Gamma Knife surgery, *NR* Not reported, *R* Retrosigmoid approach, *TL* Translab approach, *CSF* Cerebrospinal fluid

## Search strategy

A search strategy evaluated the Medline and Embase databases to search for articles published from 1968 to June 2023. The following MeSH terms or combination of those were used either in title/abstract: “radiosurgery” AND “vestibular schwannomas” AND “large” OR “Koos IV” AND “subtotal” OR “Partial” AND “surgery” or “microsurgery.”

## Inclusion criteria

We included prospective and retrospective studies, written in the English language, that reported on patients treated with a hybrid microsurgery-SRS approach. Outcomes of interest consisted of tumor control and cranial nerve preservation rates. Only those studies reporting these outcomes were included for final analyses. Concerning the pre-surgical tumor definition, some of the reviewed studies used Koos grading [18] while others used the Hannover classification [15]. In the present analyses, we allowed entries following either classification systems. Surgical details were noted including the type of surgical approach, the type of resection (partial, subtotal, etc.), cranial nerve outcomes, and other complications. SRS details were also noted including the prescribed physical dose. We retained 13 series [7, 19–29]^,49^, reporting on the outcomes of 677 patients, for pooled analysis.

## Study exclusion

A minimum of three patients had to have been reported upon, and those studies exclusively specific to patients with neurofibromatosis type II were excluded [30]. Duplicate studies identified were treated as follows: of two studies by Radwan et al. [26, 31], reporting outcomes for the same number of patients and institution, only the latest publication with updated follow-up (2021) was retained. A similar approach was used for those reports consisting of updated follow-up for the same cohorts by Daniel et al. [19, 32] and Iwai et al. [20, 21, 33].

## Radiosurgery technique

Most patients were treated with single fraction SRS. However, in a minority of cases hypofractionated SRS performed after microsurgery. For example, in the series from Radwan et al. [31], hypofractionated SRS was chosen for patients who had recovery from a cranial nerve deficit lasting longer than 3 months and/or a residual volume more than 3 cc. Details pertinent to the dosimetric data are summarized in Table 2.

**Table 2:** Outcomes: vestibular schwannoma local control and cranial nerves preservation rates for patients with large vestibular schwannomas treated with planned subtotal resection and SRS; tumor increase after SRS and post-SRS complications

References	Tumor diameter at surgery	Tumor volume at surgery	Postsurgery facial outcome	Postsurgery cochlear outcome	Interval surgery and SRS (mths)	Tumor size at SRS (mm)	Tumor volume at SRS (mL)	Marginal dose (Gy)	Tumor control; stability; decrease after SRS	Tumor increase after SRS	Post-SRS complications
Iwai et al.[21] (2003)	30–40:6/14 40–50:6/14 > 50:2/14	NR	I: 10/14 II: 2/14 HB I or II: 12/14 III:2/14 HB I or II: 10/14 III or more: 4/14	1/3	2.9 (-;1–6)	18.9(-;9.8–36.1)	–	12.1 (;10–14.1)	11/14 (78.6%) 5/14 (3.6%) 6/14 (4.3%)	3/14 (21.4%) One NF2 pt underwent salvage MS	0/14 (0%) HB I/II: 12/14 (85.7%) (stable compared to surgery) Cochlear 1/3, 33.3% (stable compared to surgery)
Park et al.[25] (2006)	35.4 (-;30–47.2)	26.8(-;13.5–55.1)	NR	NR	-(-;0.25–6)	NR	4.6(-;-)	12	8/8 (100%);-;-	0/8 (0%)	0/8 (0%) HB I/II 7/7 (100%) Cochlear NR
Fuentes et al.[49] (2008) not reviewed	39.4 (-;35–45)	NR	7/8	NR	9(;6–12)	18(-;9–20)	1.2(-;0.3–2.2)	11.8(-;11–13)	8/8 (100%);-;-	0/8 (0%)	0/8 (0%) HB I/II: 7/8, 87.5% (stable compared to surgery) Cochlear NR
Yang et al.[29] (2008) not reviewed	NR	20.6 (-;4.1–44.5)	HB I or II: 58/61	5/10	5.8 (-;0.3–95.7) Starting 2000, between 4–6 months after surgery	NR	NR	12.5 (-;9–14)	58/61 (95.1%) 8/61 (13.1%) 50/61 (82%) 8y: 93.5%	3/61 (4.9%)	0/61 (0%) 58/58 (100%) HB I/II 3/5 60%) kept serviceable hearing after SRS 3/10 (30%) if considering combined
Van de Langenberg et al.[28] (2011)	35(-;26–54)	14.9 (-;4.1–36.1)	HB I/II: 34/50 III or more: 16/50	1/4	8.5 (-;2–24)	NR	3.34(-;0.22–11.8)	11(-;9.4–11.9)	45/50 (90%) 16/50 (32%) 29/50 (58%)	5/50 (10%) Second GKS: 3/50 (6%) Second MS + SRS: 1/50 (2%)	HB I/II: 47/50, 94%(13 had transient deficit after surgery) 2/50, 4% had transient facial nerve deficit 1/50, 2% persistent hemifacial spasm 1/4, 25% same as after surgery
Pan et al.[24] (2012)	NR	G1: 17.5 ± 1.1 G2: 16.4 ± 0.95	G1: HB I/II: 16/18 G2: HB I/II/III/IV: 2/4/3/8	G1: 11/11 G2: 0/11	G1: 3.6 ± 0.2 G2: 7 ± 0.4	NR	G1: 9.35 ± 1.02 G2: 1.1 ± 0.14	G1:12(12;-) G2: 12(12;-)	G1: 18/18 (100%) NR NR G2: 17/17 (100%)	G1: 0/18 (0%) G2: 0/17 (0%)	18/18, 100% (2 recovered distantly from a facial palsy after surgery) G1: HB I/II: 16/2 Cochlear: 11/11 CSF shunt: 1 G2: HB I/II/III/IV: 2/4/4/7 Cochlear: 0/11 CSF shunt: 2
Daniel et al.[19] (2018)	33(31.5;20–45)	11.8 (-;1.5–34.9)	HB I: 47/47 Including 1 recovery (IV to I)	19/22	6(5;3–13.9)	NR	3.3 (-; 0.5–12.8)	12 (12;11–12)	43/47 (91.5%)	4/47 (8.5%) 3 Microsurgery and 1 unknown details	HB II: 1/47 (2.1%) 3 years after SRS HB I: 46/47 (97.9%) 19/22 (86.4%) hearing preservation as after surgery
Troude et al.[30] (2019)	Extrameatal 30.2(30;19–55)	16.5(14;4–87)	HB I: 122/145 HB II: 22/145 HB I/II: 144/145 HB IV: 1/145	2/19 preserved	6.8 (-;3–11)	NR	0.83(0.55;-)	12(12;-)	62/77 (80.5%)	15 regrowth (1 FU lost, 4 Observation, 9 GKS and 1 Microsurgery)	HB I/II: 144/145 (99.3%) same as after surgery 2/19 (10.5%) cochlear preservation, same as after surgery
Molina et al.[27] (2019) Only after tumor progression	NR	-(1.4;0.06–35.8)	NR	NR	-(49;2–315)	NR	- (1.4; 0.06–35.8)	- - (13; 12–14)	136/158 (86.1%)	22/158 a(13.9%) after a median of 26 mths (5–56) 14/148 (9.5%) clinical with second SRS (6/148, 4%) or microsurgery (8/148, 5.4%)	At median 36 (3–180) Trigeminal: 3/148 (2%) Facial: 3/148 (2%) Cochlear: 2/149 1.3%)
Ng et al.[23] (2020)	NR	NR	HB I or II: 6/10 HB III: 3/10 HB VI: 1/10	NR	41.8 (23.7; 3.6–117.5)	14.9 (15.7;3.9–26.8)		12–13 Gy at the 50% isodose line	8/10 (80%) 7/10 (70%) 1/10 (10%)	2/10 (20%) at 23 months after SRS (increased by 23% and by 37% respectively)	HB I/II: 7/10 (70%) HB III: 2/10 (20%) HB V: 1/10 (10%) Cochlear NR
Radwan et al.[31] (2021) not reviewed	Maximum diameter > 40 mm	13.1(-;-)	Immediate after surgery HB I-III: 15/22 HB IV-V: 5/22 HB VI: 2/22		9.5 (7, 2–50)		2.9(-;-)	10/17 single fraction 12–14 Gy 7/17 multisession (25 Gy in 5 fractions or 21 Gy in 3 fractions)	14/17 (82.4%) 13/17 (76.5%) 1/17 (5.9%) Radiological control: 80% Oncological control: 100% Mean extent of resection was 77%	3/17 (17.6%)but not requiring treatment	HB I/II: 19/22 (86.4%) same as after surgery 7/8 87.5%), after surgery 8/8 (100%) 1/8 (12.5%) declined after SRS
Iwai et al.[20] (2021) not reviewed	-(32;25–52)	NR	HBI/II: 44/47 HB IV: 1/47 HB V: 2/47	Improved: 2/16 Preserved: 13/16	-(3;1–12)	NR	-(2.7; 0.4–10.4)	-(12;10–12)	43/47 (91.5%) 3y: 92% 5y: 86% 10y: 86% 15y: 86%	4/47 (8.5%) After a median of 31 months (12–42) after SRS	HB I/II: 44/47 (93.6%) same as after surgery Cochlear 13/16 (81.3%) 0/47 (0%) ARE 2/47(4.2%) transient hemifacial spasm 2/47 (4.2%) transient trigeminal neuropathy associated with TTE
Lee et al.[22] (2021)	NR	-(15.4;3.2–40.9)	HB I/II/III/IV/V: 39/15/6/7/1	6/27 preserved	(4.2;0.7–16.2)	NR	-(2.5;0.3–27.4)	-(12.5;10–20) 1 case underwent fractionated GKS 20 Gy in 4 fractions	60/68 (88.2%)	8/68 (11.6%) after a median progression time of 15.8 mths (3.2–66)	HB I/II/III/IV/V::44/13/2/7/2 (4 recovered between surgery and SRS; 2/68 (3%) aggravated facial palsy from HB II) 6/27 (22.2%) preserved, as after surgery Others: 3/68 (4.4%) hemifacial spasm

*SRS* Stereotactic radiosurgery, *HB* House et Brackmann classification, *NR* not reported, *MS* Microsurgery

## Outcomes

The primary outcome variable for this analysis was crude tumor control, defined as either stability or a decrease in tumor volume compared to baseline measurements. Secondary outcomes included functional preservation rates for the facial and cochlear nerves. Most studies used the House-Brackmann (HB) scale [34] for facial nerve assessment, and the Gardner-Robertson (GR) scale [35] for cochlear nerve-hearing classification. A favorable facial outcome was considered in the majority of included studies as HB I or II. A favorable hearing outcome (i.e. cochlear functional preservation) was considered in the majority of included studies as GR class 1 or 2 (functional hearing). Post-operative trigeminal dysfunction was also noted in the postoperative analyses.

## Statistical analysis

Because of high variations in study characteristics, a statistical analysis using a binary random-effects model (DerSimonian–Laird method) was performed using OpenMeta analyst software (Agency for Healthcare Research and Quality). Weighted summary rates were determined using meta-analytical models. Heterogeneity was tested for each meta-analysis; pooled estimates were obtained for all outcomes. The results of series concerning the local control and cranial nerve morbidity outcomes were pooled using a meta-regression with a random effect. P values < 0.05 were considered statistically significant.

## Results

### Included studies and patients

Demographic data are summarized in Table 1. The criteria and strategy for a combined approach was heterogeneous among studies; most considered a Koos grade IV or a tumor with an extrameatal diameter of more than 30 mm as candidates for microsurgery-SRS [36]. The follow-up period was variable, but with a minimum mean of 32 months and ranged from 11–180 months. The most commonly reported microsurgical resection outcome was a STR. Postoperative complications (others than related to facial and cochlear functions) are summarized in Table 1.

## Tumor control

**Fig. 2:**
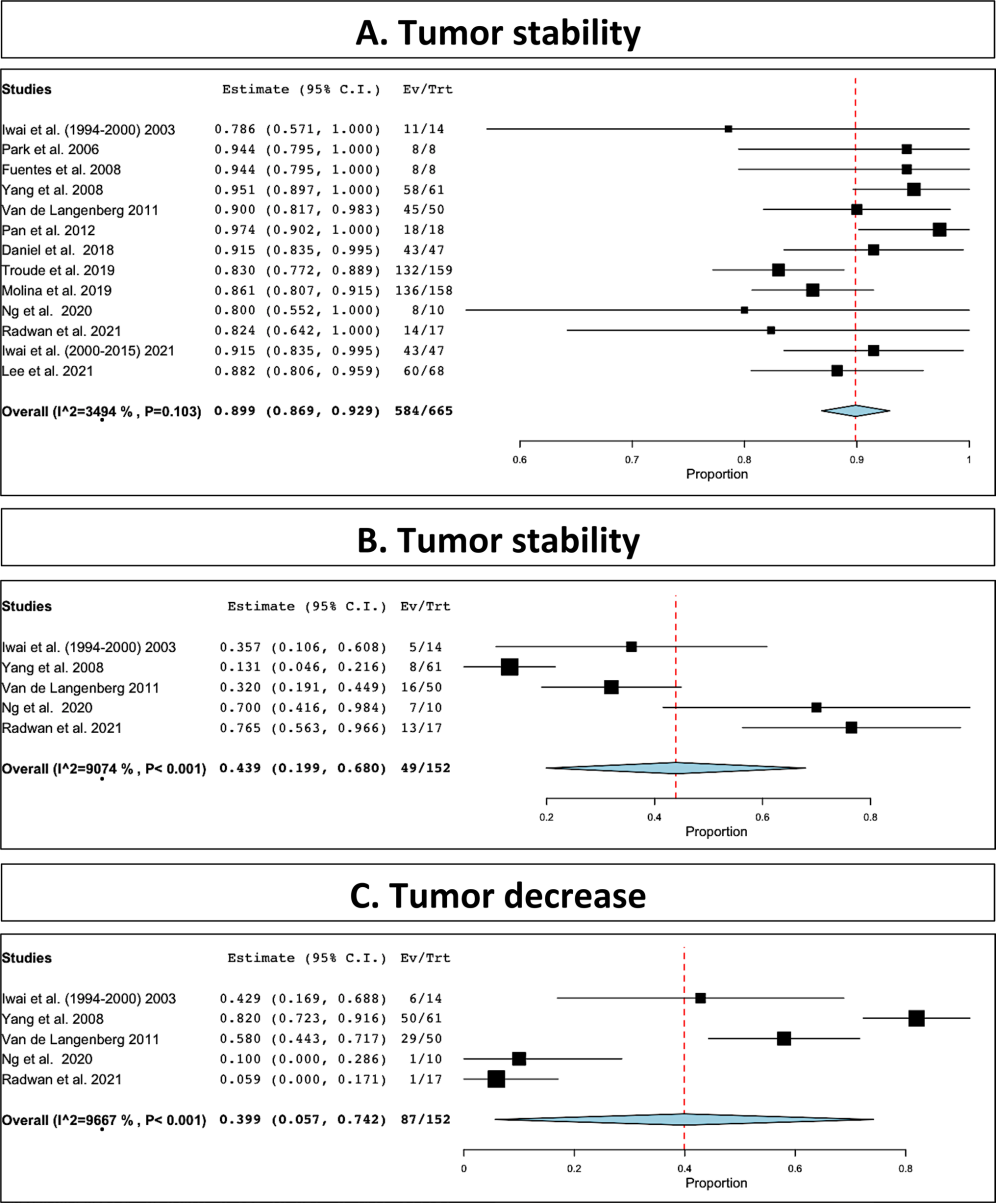
Tumor control: **A**, stability and decrease included; **B**, stability; **C**, decrease

Tumor control was reported in only 665 patients. In 584 tumor was considered stable or reduced (89.9%, range 86.9–92.9%, I^2^ = 34.9, p heterogeneity = 0.103, *p* < 0.001; Fig. 2A). Tumor stability (reported for only 152 patients) was observed in 49 patients, 43.9% (range 19.9–68%, I^2^ = 90.74, *p* heterogeneity < 0.001, *p* < 0.001; Fig. 2B), and tumor reduction (reported for only 152 patients) was observed in 87 patients, 39.9% (range 57–74.2%, I^2^ = 95.43, *p* heterogeneity < 0.001, *p* = 0.02; Fig. 2C).

## Functional preservation

**Fig. 3:**
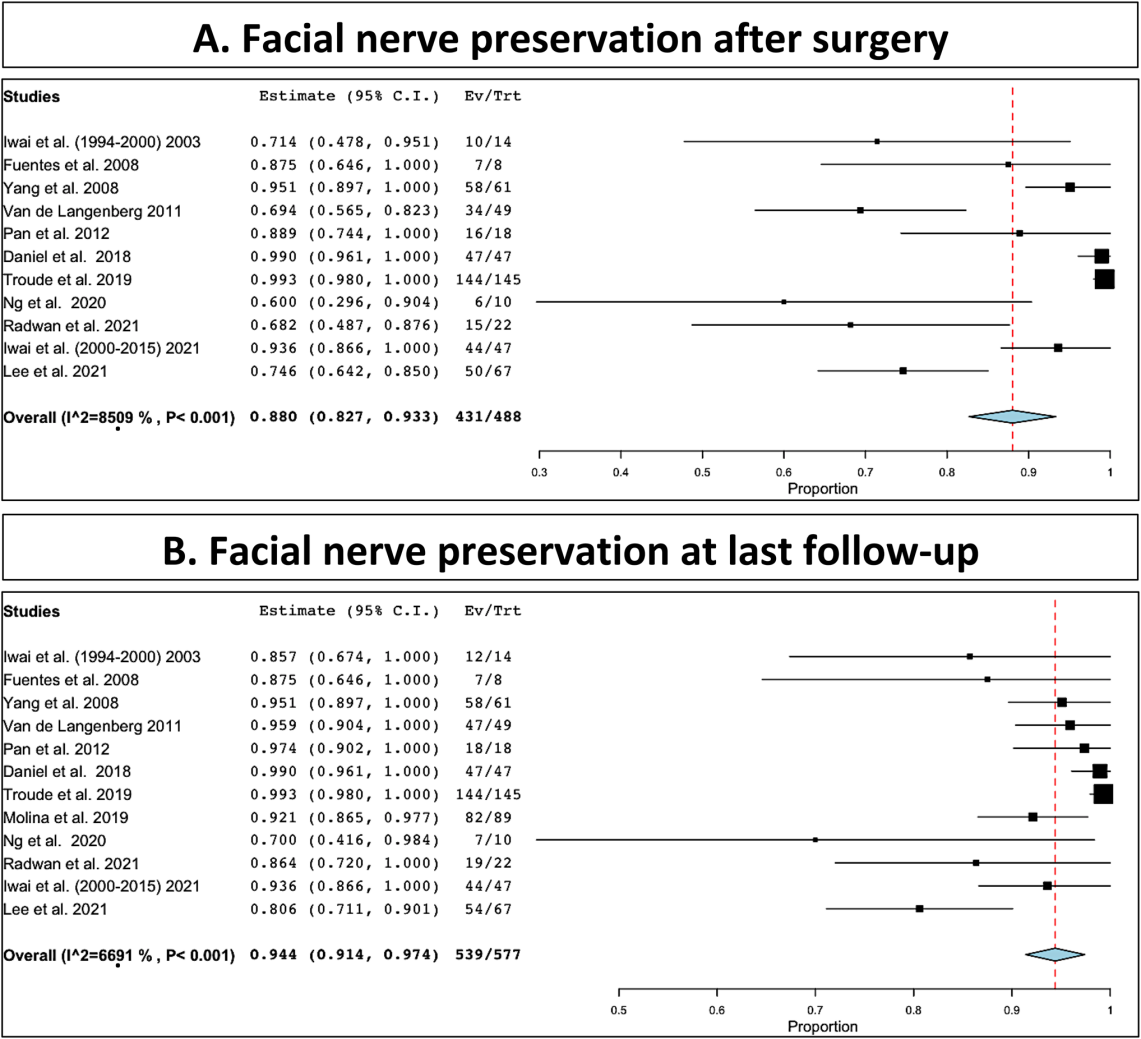
Facial nerve preservation: **A**, immediate after surgery; **B**, at last follow-up

**Fig. 4:**
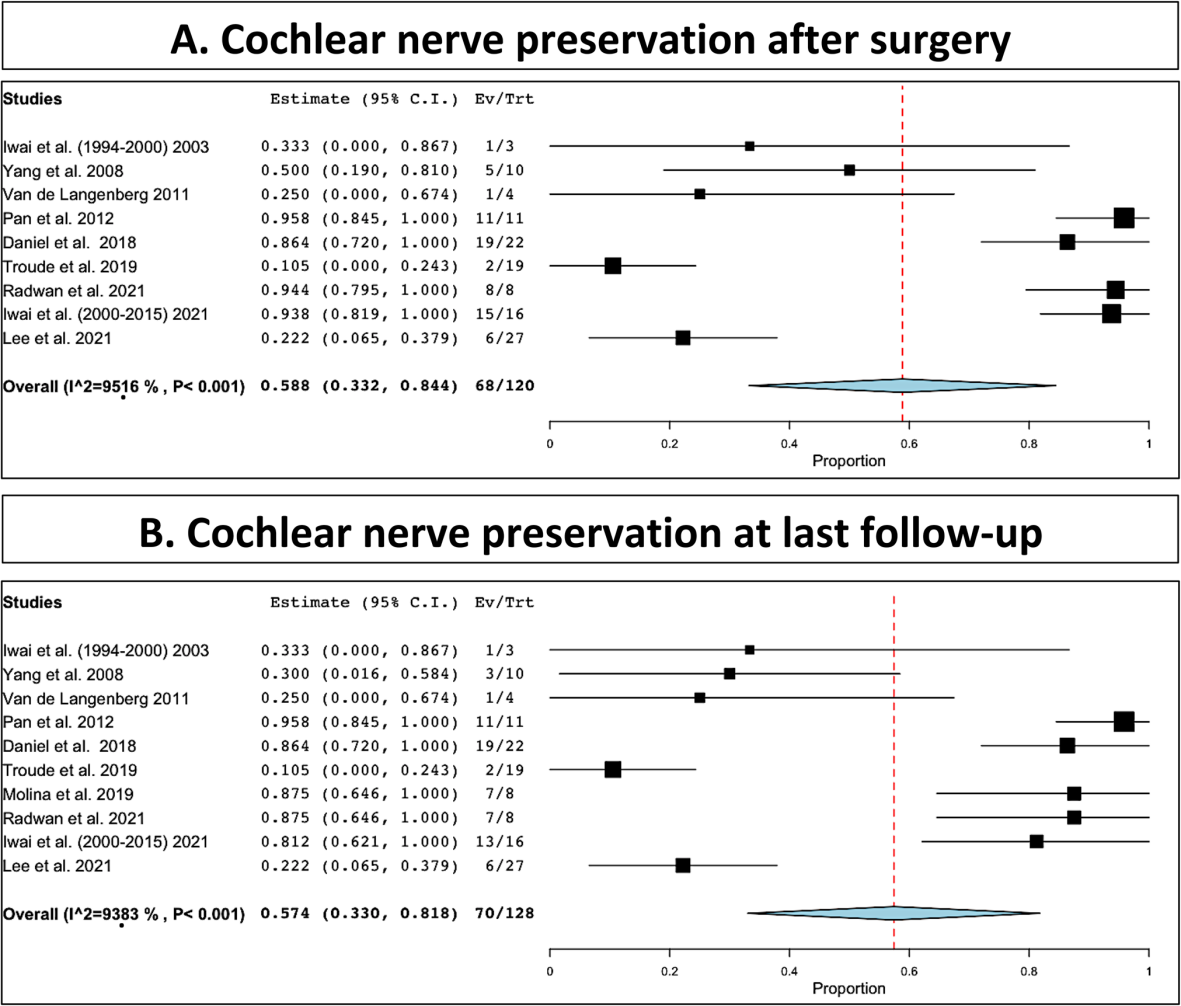
Cochlear nerve preservation: **A**, immediate after surgery; **B**, at last follow-up

Facial nerve functional preservation after microsurgery was observed in 431 out of 488 patients (88%, range 82.7–93.3%, I^2^ = 85.09, p heterogeneity and p < 0.001; Fig. 3A). Facial nerve functional preservation at last follow-up was 539 out of 577 patients (94.4%, range 91.4–97.4%, I^2^ = 66.91, p heterogeneity and p < 0.001; Fig. 3B). Cochlear functional preservation after surgery was 68 out of 120 patients (58.8%, range 33.2–84.4%, I^2^ = 95.16, p heterogeneity and p < 0.001; Fig. 4A). Cochlear functional preservation at last follow-up was 70 out of 128 patients (57.4%, range 33–81.8%, I^2^ = 93.83, p heterogeneity and p < 0.001; Fig. 4B, Table 3).

**Table 3:** International Stereotactic Radiosurgery Society Practice Recommendations for management of large vestibular schwannomas with combined microsurgery and stereotactic radiosurgery

Recommendation category	Practice recommendation	Strength of recommendation	Level of evidence
Principles of microsurgical surgical resection	Nerve sparing intracapsular microsurgical resection with facial nerve preservation	Strong	III
Post-operative SRS	Upfront SRS should be considered as opposed to observation due to the potential to optimize long-term local control and within 3–6 months following STR	Conditional	III
RT alone	RT alone can be used as upfront treatment in patients with medical comorbidities precluding upfront surgery	Strong	III
Monitoring and follow-up recommendations for toxicity evaluation	A first post-operative MRI should be performed within 3–6 months to determine residual disease and an evaluation for post-operative SRS	Strong	III

*MRI* Magnetic resonance imaging, *SRS* Stereotactic radiosurgery, *RT* Radiotherapy

## Discussion

Our systematic review and meta-analysis suggest that a combination of microsurgery-SRS achieves high rates of tumor control and an highly satisfactory facial nerve functional preservation. Cochlear functional preservation was adequate at 57.4% at last follow-up. The limitations and quality of the data reporting prevented a more detailed analyses, as such, we learned much from the individual papers to address key questions to guide our understanding of hybrid surgery-SRS.

## General considerations

In the management of large VS, one has to balance the intent of tumor control and functional preservation of the cranial nerves when determining the optimal treatment strategy. The appealing concept of intracapsular removal, suggested recently as suitable for combined approaches, is not new. Cushing himself advocated for intracapsular removal, which was further demonstrated to have high progression rates [37]. Furthermore, Mckenzie et al. [38] suggested excellent results with such an approach, with low incidences of lower cranial nerve dysfunction, brainstem injury, or facial palsy. Lownie et al. [39] reported radical intracapsular removal in 11 patients, with facial nerve preservation in 82% of patients. In summary, intracapsular removal can reduce operative morbidity while preserving neurological function. However, it does not ensure long-term tumor control [40], which has raised the interest of SRS as an immediate adjunctive treatment to the residual [41]. Iwai et al. pioneered the concept of intracapsular removal followed by SRS in order to increase cranial nerve preservation while keeping high rates of tumor control, with a first patient treated as early as 1994 [21].

## Factors involved in better tumor control in the context of combined approaches

The probability of local control to residual disease following microsurgical resection might have different underlying mechanisms due to devascularization and tumor hypoxia [42–44]. It may be that the smallest volume of residual disease achievable while sparing the surrounding cranial nerves is the goal. Iwai et al. [20] suggested that treatment failure was associated with lesion volume before irradiation (with a cut-off of 6 cc), with a trend for higher incidence of larger tumors in younger patients. Lee et al. [22] noted that residual postoperative tumor volume was associated with tumor progression in both uni- and multivariate analysis (cut-off of 6.4 cc); patients with lower residual tumor volumes were associated with better 5-year progression free survival rates. Other authors confirmed such findings, suggesting that in order to achieve long-term tumor control, tumor volume with single fraction SRS should be smaller than 6 cc [33]. In a previous study [45], reviewing the outcomes for upfront single fraction SRS for large VS, the cut-off for better tumor control was 10 cc. The likely lower cut-off for tumor control, suggested here, might open the discussion of a more aggressive biology in such patients with large VS needing initial surgery.

## When to perform SRS: Early versus late

Data on the exact timing of SRS after microsurgical resection are scarce and subject to considerable practice variation. Some advocate that only GTR patients or those with a minimal residual could potentially be “devascularized”, and do not need immediate adjuvant SRS [7]. However, the same group acknowledged that tumor remnants are likely to grow in the future [7]. Ng et al. [23] retrospectively divided patients in 2 groups after STR: an early intervention (i.e. SRS) group and an observation/delayed group, and confirmed the safety of this approach in both groups. The early intervention group consisted of patients having SRS within the first 12 months after surgery, with either sizable residual tumor of more than 1 cm, a rapidly growing tumor (more than 2 mm per year), or patients who preferred to receive early treatment with SRS. The authors concluded that SRS of residual or recurrent tumor is safe following STR and appears to carry a low risk of worsening facial nerve function when performed for progressive tumor growth.

Iwai et al. [20] suggested performing SRS 3 months after the microsurgical resection, since by that time, postoperative changes should have regressed and the shape of the lesion would be stable for SRS. The same authors suggested that in case of cystic tumors, SRS should be performed as early as one month after surgery, to avoid cyst reexpansion, enlargement of target volume, and potential for further cyst changes as a result of SRS [20]. Radwan et al. [26] suggested that facial nerve function did not differ significantly on the basis of the irradiation timing (early versus late) or on the basis of the irradiation regimen (single-session versus multisession). In summary, in cases of subtotal resection, we suggest performing stereotactic radiosurgery as an adjuvant treatment within 3–6 months after surgery. This has been previously suggested by Daniel et al., as the open residual capsule will progressively folds on itself, due to brain pulsations and volumetric reduction of the residual tumor [32].

In summary, the literature suggests that early SRS should be considered as opposed to observation of the residual tumor due to the potential to optimize long-term local control. We are unable to confidently define a volume of residual that may be optimal to observe.

## Cranial nerve outcomes

Facial nerve outcome is of major importance for maintaining postoperative quality of life [46, 47]. In patients with large tumors, the prevalence of excellent-to-good HB grades I and II after surgery averages 54% (range, 44% to 94%) [26]. Schwartz et al. [48] reported that better facial nerve function preservation and lower incidence of HB grade VI may be achieved after NTR (78% vs. 2% respectively) or STR (71% vs. 10% respectively) of a large VS in comparison with GTR (53% vs. 24% respectively). Park et al. [25] advocated that facial nerve preservation was inversely proportional to the extent of removal and tumor volume, thus suggesting that incomplete removal might improve the chance of preserving facial nerve function as compared to complete removal. Moreover, patients with larger preoperative VS had lower chances of facial nerve preservation. The rates of facial nerve preservation after microsurgical resection of large VS are also a function of the microsurgical approach and the quality of tumor resection [49]. The postoperative HB grade inversely correlated with the residual tumor volume, with the best outcomes generally attained in cases with residual tumor volumes of more than 3 cc[26].

Preservation of functional hearing after total removal of large VS is difficult, and hearing preservation ranges between 5 to 56% [20, 50–53]. This range of outcomes conveys that hearing preservation after surgical resection is still more difficult compared with the effort to preserve the facial nerve. Hearing deterioration was reported to range between 22.2%-29.4% via the retrosigmoid approach [54, 55] and in 56% of patients after the middle fossa approach at 5 years follow-up [52]. There are reports suggesting the possibility of hearing improvement after microsurgery [52, 56], while relevant factors were preoperative good hearing level, poor speech discrimination, small tumors, short-term onset of hearing loss, and superior vestibular nerve origin [52].

In our review of combined approaches, only a few studies have reported on the preservation of the cochlear nerve function. However, each of the studies individually and all of them collectively appear to demonstrate that STR followed by SRS seems to have a better outcome in terms of preservation of hearing compared to the results reported in series with resection alone. Indeed, when reported, hearing preservation after resection was 57–58% and could be maintained even after SRS.

Upfront SRS for large VSs.

Although large vestibular schwannomas are traditionally considered an indication for microsurgical resection, upfront single-fraction stereotactic radiosurgery (SRS) may be a viable option for select patients (Class C evidence). When comparing outcomes between patients without prior surgery and those who had undergone previous surgical intervention, studies have shown higher tumor control rates, greater tumor reduction, lower likelihood of requiring additional treatment (microsurgery, shunt placement, or SRS), increased incidence of new-onset facial palsy (though still relatively low), and improved hearing preservation rates (Class C evidence) [45].

## Limitations

Our meta-analysis has several inherent limitations due to the lack of uniformity in the reporting of surgical, SRS and patient selection factors.

## Treatment intention

Some studies included patients that were not pre-selected for STR and SRS. Some patients with GTR followed by recurrence may be included and these would likely add negative bias to the results.

## Surgical factors: heterogeneity of surgical techniques

Heterogeneity of the surgical technique, including the surgical approach, opening or not of the internal acoustic meatus, etc., might have influenced the results. In some series, such as the one by Gupta et al. [57], it was difficult to extract data on local control with regards to the patients who underwent microsurgery and SRS, as these have been only scarcely reported within the manuscript. Some studies [27] also included patients with multiple surgeries on the same tumor.

## Radiation factors: heterogeneity of irradiation techniques

Although most studies reported the use of single fraction session SRS, one did include staged-volume radiosurgery for a large postoperative residual tumor[21]. Some series included a mix of single fraction and hypofractionated SRS, which could be associated with a different treatment effects in terms of radiobiology [26].

## Toxicity assessment and follow-up

There was a variability in the use of outcome measure scales. Indeed, some authors [1] determined cranial nerve outcomes using chart reviews, which is subject to underreporting as compared to the direct clinical assessment. Not all studies included both the PTA and speech discrimination, leading to additional bias in hearing evaluation.

Prior surgical tumor volumes were not measured in all studies, making the comparison pre- and postoperative challenging [27]. Criteria for a decrease in tumor volume was variable given the lack of consensus guidelines for tumor response assessment; Iwai et al. [33] considered such if there was a decrease of the longest dimension by more than 30%. Also, criteria for combined approaches with regards to linear and/or volumetric measurements remains extremely heterogeneous. S

## Patient selection factors

Another limitation is related to the heterogeneity of patients, as several studies included patients with diagnosed type II neurofibromatosis, which is now acknowledged to have different tumor control rates as compared to sporadic VS. There was also a lack of detailed data on pre-operative hearing status.

## Data reporting

Definition of tumor control and cranial nerve outcomes as crude rates, instead of 1-, 5- and 10-year time-dependent actuarial outcomes, which are missing. Such is related to multiple heterogeneity sources, which cannot be controlled, and derive both from a variety of practices among centers and the retrospective data collection. Moreover, no series reported Kaplan–Meier estimators. In this respect, actuarial outcomes in future series are needed in order to help defining time-dependent outcomes for pooled series.

Detailed definitions of tumor size should be provided, in mm and Koos grade class. Universally accepted reported outcomes scale, such as the House-Brackmann for the facial nerve and the Gardner Roberson for hearing should be used. Moreover, the definitions of STR and GTR should be provided in further manuscripts, based upon the percentage of resection.

## Conclusions

A cranial nerve sparing approach with subtotal microsurgical resection and SRS to the residual tumor achieves high rates of tumor control (as high as 90%). Using such a combined approach, facial nerve preservation at last follow-up was 94.4% (better compared to the immediate postsurgical period) and cochlear functional preservation at last follow-up was 57.4%. Combined approach with subtotal resection followed by SRS for large VSs appears to be relatively safe management strategy which yields good tumor control and an acceptable level of cranial nerve preservation, by a nerve sparing approach.

Based on the previous, we could sum several recommendations (Table 3, level III evidence): nerve sparing intracapsular microsurgical resection with facial nerve preservation; a first postoperative MRI should be performed within 3–6 months to determine residual disease and an evaluation for postoperative SRS; consider postoperative upfront SRS as opposed to observation due to potential to optimize long-term control and within 3–6 months after The most commonly reported microsurgical resection outcome was a STR.; radiotherapy alone can be used as upfront treatment in patients with medical comorbidities precluding upfront surgery;

## Data Availability

No datasets were generated or analysed during the current study.
